# Cognitive Motor Dual Tasking as a game-changer in basketball training programs: a new approach to developing elite basketball players

**DOI:** 10.3389/fpsyg.2025.1665556

**Published:** 2025-11-11

**Authors:** Asaf Shalom, Roni Gottlieb, Daniele Conte, Julio Calleja-Gonzalez

**Affiliations:** 1 Department of Physical Education, The Research Center for Sports and Physical Activity, Tel Hai College, Upper Galilee, Israel; 2 Wingate Institute, The Academic College Levinsky-Wingate, Wingate Campus, Netanya, Israel; 3 Department of Coaching Science, Lithuanian Sports University, Kaunas, Lithuania; 4 Department of Movement, Human and Health Sciences, University of Rome “Foro Italico”, Rome, Italy; 5 Department of Physical Education and Sports, Faculty of Education and Sport, University of the Basque Country, EHU, Vitoria-Gasteiz, Spain

**Keywords:** basketball, Key Performance Indicators, Cognitive Motor Dual Tasking, technology, training program

## Introduction

In basketball, as an open-skill sport, coordinating physical, technical, and mental components is a key challenge in the training process (Shalom et al., 2023; Zhang et al., 2025). A deep understanding of the game's specific demands, combined with physiological and biomechanical analyses of movement, is essential for the accurate planning of team training programs (Sosa et al., 2025). Such understanding enables the making of informed decisions when selecting appropriate training methods, ensuring targeted preparation for both practices and competitions, integrating advanced technologies, and designing tailored exercises using specialized equipment (Gottlieb et al., 2021; Marocolo and Souza, 2024). Although the past decade has seen significant progress in the integration of physical and cognitive components, along with the implementation of advanced technologies in elite sports, when considering basketball training programs, a substantial gap remains between the strong focus on physical training and the limited attention given to specific, on-court cognitive training (Fuster et al., 2025). This is despite the growing recognition of the importance of integrating both the physical and cognitive demands of the modern game (Zhang et al., 2025). Indeed, basketball requires rapid decision-making and, real-time adaptations to changing situations, and immediate responses to continuous pressure from various factors such as opponents and environmental (e.g., crowd noise) and variable playing conditions (Lucia et al., 2021; Shalom et al., 2025).

This opinion article presents a modern approach that places cognition at the center of the planning and design of training programs, aiming to reduce the gap between physical performance and the cognitive demands of high-level basketball. Such approach has the potential to enhance specific Key Performance Indicators (KPIs) relevant to elite level performance, while also highlighting the potential for applying advanced integrative technology (Beals and Paris, 2024) in the particular context of basketball.

## Cognitive Motor Dual Tasking in action: on the basketball court

Scientific literature has shown that when performance staffs (Calleja-González et al., 2021) assess the specific demands of basketball to optimize KPIs relevant to elite level competition, they consistently observe that the game is characterized by repeated short-duration, high-intensity, and dynamic actions (Gottlieb et al., 2021). These actions require a high level of explosive power, which relies primarily on anaerobic energy systems—most notably, the alactic system (Shalom et al., 2023). Simultaneously, the aerobic system plays a critical role in recovery between efforts, allowing players to maintain performance under sustained physical demands (Gottlieb et al., 2023; Martinho et al., 2025).

However, in addition to physiological demands, modern basketball also challenges players with complex cognitive demands that require control of essential psychomotor components such as reaction time, agility, coordination, kinesthesia, and balance (Habay et al., 2021; Lakhno et al., 2020). Recent research and developments highlight the growing importance of cognitive training, including in other sports and in injury rehabilitation, where psychomotor skills often decline (Brinkbäumer et al., 2024; Jiménez-Martínez et al., 2025; Wu et al., 2024). Therefore, a deep understanding of the interaction between physiological and cognitive requirements is essential for designing effective short- and long-term training programs that aim to enhance performance in real competitive environments (Shalom et al., 2023).

Many training approaches focused on developing explosive power through closed-skill tasks such as short sprints, sharp changes of direction, vertical jumps, and complex jump patterns involving both vertical and horizontal components (Cormier et al., 2020; Rodríguez-Rosell et al., 2017). These types of exercises are easier to measure and assess, and are relevant to other team sports in addition to basketball (Alba-Jiménez et al., 2022; Gottlieb et al., 2021).

Nevertheless, to the best of our knowledge, the scientific literature remains limited when it comes to enhancing explosive power within open-skill contexts such as agility, or when encompassing sprinting and jumping tests that involve responses to unpredictable stimuli. In this sense, research has yet to provide sufficient frameworks for training methodologies that reflect the true cognitive motor demands of game situations (Zhang et al., 2025).

Over recent decades, many brain training approaches have been developed—mostly based on computer software and mobile applications. While many of these tools show improvements in specific tasks, their real-world transferability is often limited (Alemanno et al., 2025; Hülsdünker et al., 2025b). In contrast, several recent studies point to clear advantages of movement-based cognitive motor training (Lucia et al., 2023b; Moreira et al., 2021). For example, one study comparing passive, computer-based brain training to active cognitive motor training found that the latter was not only more effective in improving cognitive skills, but also led to positive changes in brain activity (Hülsdünker et al., 2025b). These findings support the idea that cognitive training programs should integrate complex motor components to enhance functional transfer to real-life performance. Another study showed that a 5-minute cognitive motor recovery session following exhaustive physical activity was more effective than passive recovery in improving reactive agility (Hülsdünker et al., 2025a).

In this context, CMDT is an integrated training method that targets both physical and cognitive skills simultaneously, and has demonstrated strong potential in enhancing basketball-specific performance (Lucia et al., 2023b, 2021). Unlike isolated physical or cognitive training, CMDT enables simultaneous improvements in explosive power, attention, reaction time, and memory within a framework that mimics open, dynamic game situations. The integration of physical exertion with cognitive demands creates more realistic and transferable training conditions, enhancing decision-making and adaptability under pressure and fatigue (Lucia et al., 2023b).

Moreover, implementing CMDT as part of basketball warm-ups has also proven effective for preparing players for explosive movements that require both closed- and open-skill responses (Shalom et al., 2025). Moreover, combining CMDT with other established methods such as Multi-Component Training (MCT) has shown great potential to increase basketball players' performances (Brunner et al., 2019; Stojanović et al., 2023). In this regard, previous research has demonstrated significant improvements in basketball-specific open-skill performance after just 5 weeks of CMDT integrated within an MCT framework (Lucia et al., 2023b).

From both research and practical standpoints, CMDT has proven to be highly engaging and motivating for basketball since challenges players mentally and physically, improving their focus, and creating a more enjoyable training atmosphere. Beyond its potential benefits on performance, CMDT may also help reduce stress during intense periods and promote a positive training environment. It further stimulates creativity among coaches and performance staff in designing varied and innovative training methods.

## When the lab meets the court: technology as a tool for performance development in basketball

In modern basketball, performance staff are increasingly integrating advanced sports technologies as a core component in enhancing KPIs at the elite level (Calleja-González et al., 2021; Shalom et al., 2024). Innovative systems now enable the collection and analysis of a broad range of physical and cognitive data, allowing for the identification of trends, tracking of player development, and the precise adjustment of training programs. Measured parameters include short sprints, vertical jumps, changes of direction, agility, reaction time, information processing speed, decision-making, and more. Each one of these parameters is essential in the dynamic environment of competitive basketball (Mancha-Triguero et al., 2019; Shalom et al., 2023).

These assessments take place both periodically, as part of a structured monitoring framework, and in real time through wearable technologies and artificial intelligence (AI)-based smart systems. This process allows for continuous, accurate monitoring of player performance during both training sessions and live gameplay (Cheng et al., 2022; Chidambaram et al., 2022; de-Oliveira et al., 2021; Glatthorn et al., 2011; Hassan et al., 2022; Hoffman, 2020; Jovanović et al., 2024; Kołodziej et al., 2018).

One of the most rapidly evolving research areas in basketball science is the integration of CMDT training directly on the basketball court (Lucia et al., 2023a,b). No longer confined to external laboratories, these technologies effectively bring the measurement equipment onto the court itself. Through interactive devices embedded in the floor, as well as advanced lighting systems and responsive sensors, a dynamic interface is created between the player and the technology while in motion. These systems interact with players in real time, respond to their actions, and present them with variable, game-relevant challenges that simulate the complexity of actual play in a specific, practical, and highly effective manner (Fuster et al., 2025; Moreira et al., 2021; Shalom et al., 2025).

The key advantage of this approach lies in its ability to train and evaluate players while in motion, under the same pace and complexity found in real game conditions. Emerging research shows that the use of such interactive technologies not only enhances engagement and measurement accuracy, but also significantly improves cognitive motor abilities. Moreover, these gains translate directly into improved real-time performance on specific KPIs that matter most at the elite level (Klatt et al., 2025; Mao et al., 2024; Romeas et al., 2016).

## Discussion

In modern basketball, developing explosive power requires more than just general strength training. It demands a focus on specific and dynamic movements that reflect real game situations, using targeted and sport-specific training methods (Shalom et al., 2023). While drills based on closed skills, such as isolated vertical jump, short sprint and change-of-direction performance, still provide value in building a strong physical and technical foundation, they are not enough on their own to prepare players for the full complexity of high-level play (Zhang et al., 2025). Although these types of drills are easier to monitor physiologically and help manage training loads more precisely, they often lack the combination of movement and cognition that defines top-level basketball performance.

At the highest levels of competition, what truly separates excellent players from the rest is their ability to produce explosive power as a quick response to unexpected situations, while also making accurate decisions under pressure (Haugan et al., 2025). A successful player is not defined by physical strength alone, but by the ability to combine high power with quick perception, psychomotor responsiveness, and cognitive control in unpredictable, open-skill environments. In this sense, cognitive performance is not a secondary element but rather a central part of developing elite level players, and accordingly, the perception of cognitive effort should be taken into account (Halperin and Vigotsky, 2024; Zhang et al., 2025).

Although technology-based training offers valuable benefits such as accurate measurement, real-time feedback, and personalized programming, it is important to remember that basketball is played by people, not machines. To ensure that learning transfers effectively into performance, training must also include scenarios that simulate human interaction, emotional stress, environmental distractions, and uncertainty. Combining CMDT-based drills that involve reacting to human movement in real time, with accurate psychomotor measurement, alongside the use of small-sided games (SSGs), can create a relevant and effective training environment (Hassan et al., 2023; Qarouach et al., 2024, 2025). However, it should be noted that SSGs, while useful, offer coaches less control over intensity and repetition volume (Li et al., 2024). This highlights the need for a balanced approach, ie, combining technological precision, CMDT drills, human movement interaction, and psychomotor stimuli while maintaining realistic, game-like conditions.

Beyond that, there is a clear need to develop tools that can assess cognitive motor performance under real physical and mental demands. Future technologies should aim to measure decision-making, response to live stimuli, and overall execution in conditions that closely simulate actual gameplay. Innovation in this area can offer critical insights for improving training design, personalizing drills, and guiding long-term player development.

The CMDT training shows high potential not only for enhancing performance in dynamic contexts, but also as a rehabilitative tool during return-to-play processes. Even when physical limitations exist, the brain can continue to be trained through relevant movement-based tasks, helping to maintain or even strengthen specific skills that are still trainable.

Future studies should explore how CMDT integrates with external factors such as crowd noise, distractions, and emotional pressure. A smart combination of open- and closed-skill training, along with creative and balanced use of integrative technology, could serve as the foundation for a reliable and innovative training model. Such a model, grounded in fields like cognition, physiology, biomechanics, and game analysis, may shape the next generation of elite basketball players.
